# 2,5-Dichloro-*N*-(3-methyl­phen­yl)benzenesulfonamide

**DOI:** 10.1107/S1600536812032023

**Published:** 2012-07-18

**Authors:** Shumaila Younas Mughal, Islam Ullah Khan, William T. A. Harrison, Muneeb Hayat Khan, Muhammad Nadeem Arshad

**Affiliations:** a Materials Chemistry Laboratory, Department of Chemistry, GC University, Lahore 54000, Pakistan; b Department of Chemistry, University of Aberdeen, Meston Walk, Aberdeen AB24 3UE, Scotland; c Questioned Documents Unit, Punjab Forensic Science Agency, Home Department, Lahore, Pakistan; d Center of Excellence for Advanced Materials Research (CEAMR), Faculty of Science, King Abdulaziz University, PO Box 80203, Jeddah 21589, Saudi Arabia

## Abstract

In the title compound, C_13_H_11_Cl_2_NO_2_S, the dihedral angle between the aromatic rings is 76.62 (10)° and the C—S—N—C linkage between the rings adopts a *gauche* conformation [torsion angle = −51.4 (2)°]. A weak intra­molecular C—H⋯O inter­action closes an *S*(6) ring. In the crystal, inversion dimers linked by pairs of N—H⋯O hydrogen bonds generate *R*_2_^2^(8) loops.

## Related literature

For related structures, see: Khan *et al.* (2011 ▶); Mughal *et al.* (2012 ▶).
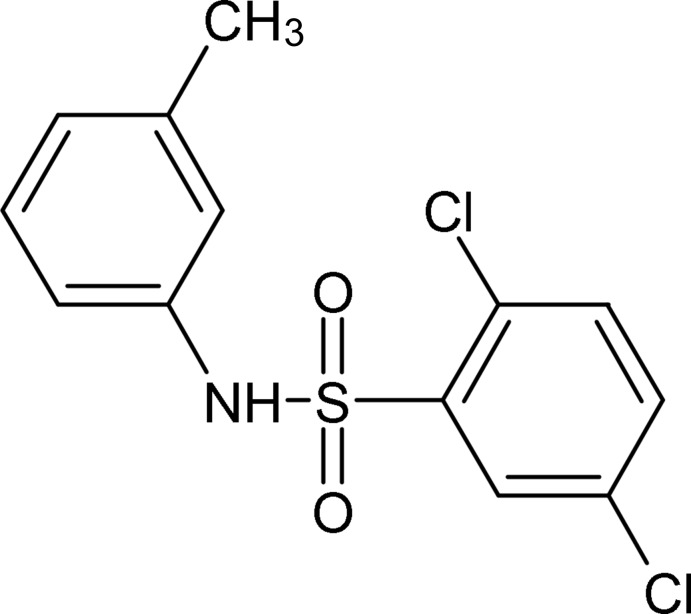


## Experimental

### 

#### Crystal data

C_13_H_11_Cl_2_NO_2_S
*M* *_r_* = 316.19
Monoclinic, 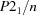

*a* = 9.0361 (10) Å
*b* = 11.6937 (11) Å
*c* = 13.6904 (15) Å
β = 100.588 (3)°
*V* = 1422.0 (3) Å^3^
*Z* = 4
Mo *K*α radiation
μ = 0.60 mm^−1^
*T* = 296 K
0.41 × 0.32 × 0.26 mm

#### Data collection

Bruker APEXII CCD diffractometer
4252 measured reflections
2566 independent reflections
2019 reflections with *I* > 2σ(*I*)
*R*_int_ = 0.033

#### Refinement

*R*[*F* ^2^ > 2σ(*F* ^2^)] = 0.052
*wR*(*F* ^2^) = 0.159
*S* = 1.06
2566 reflections
173 parameters
H-atom parameters constrained
Δρ_max_ = 0.55 e Å^−3^
Δρ_min_ = −0.46 e Å^−3^

### 

Data collection: *APEX2* (Bruker, 2007 ▶); cell refinement: *SAINT* (Bruker, 2007 ▶); data reduction: *SAINT*; program(s) used to solve structure: *SHELXS97* (Sheldrick, 2008 ▶); program(s) used to refine structure: *SHELXL97* (Sheldrick, 2008 ▶); molecular graphics: *ORTEP-3* (Farrugia, 1997 ▶); software used to prepare material for publication: *SHELXL97*.

## Supplementary Material

Crystal structure: contains datablock(s) global, I. DOI: 10.1107/S1600536812032023/xu5591sup1.cif

Structure factors: contains datablock(s) I. DOI: 10.1107/S1600536812032023/xu5591Isup2.hkl

Supplementary material file. DOI: 10.1107/S1600536812032023/xu5591Isup3.cml

Additional supplementary materials:  crystallographic information; 3D view; checkCIF report

## Figures and Tables

**Table 1:** Hydrogen-bond geometry (Å, °)

*D*—H⋯*A*	*D*—H	H⋯*A*	*D*⋯*A*	*D*—H⋯*A*
N1—H1⋯O1^i^	0.86	2.09	2.946 (3)	178
C12—H12⋯O2	0.93	2.50	3.141 (3)	126

Symmetry code: (i) 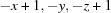.

**Fig. 1.**
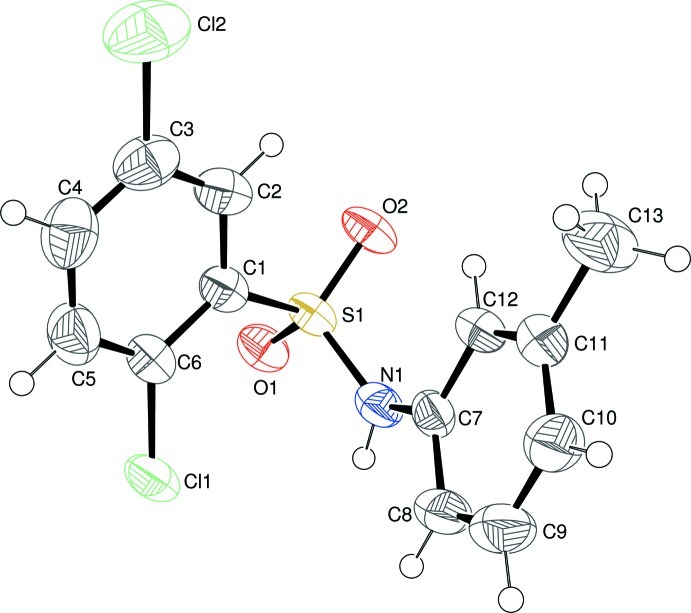
The molecular structure of (I), showing displacement ellipsoids at the 50% probability level.

**Fig. 2.**
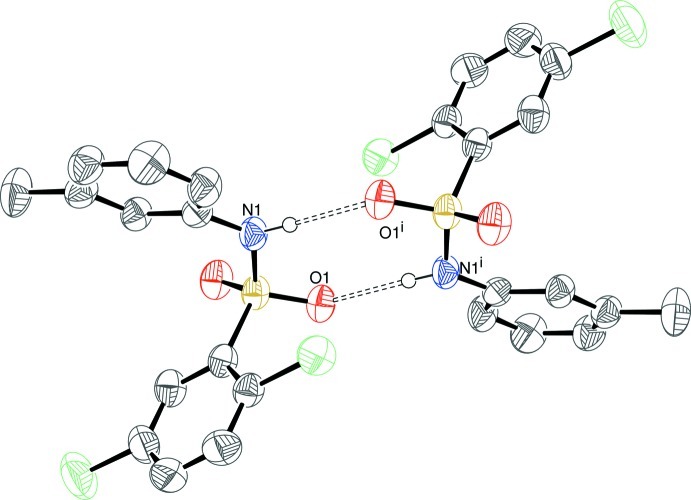
An inversion dimer in the crystal structure of (I). Hydrogen bonds are shown as double-dashed lines and all C-bound H atoms are omitted for clarity. Symmetry code: (i) 1–x, –y, 1–z.

## supplementary crystallographic information

### Comment

The title compound, (I), (Fig. 1) was prepared and characterized in continuation of our interest in the structural chemistry of sulfonamides (Khan *et al.*, 2011; Mughal *et al.*, 2012).

The dihedral angle between the C1—C6 and C7—C12 benzene rings is 76.62 (10)°. The C1—S1—N1—C7 linkage between the rings adopts a *gauche* conformation [torsion angle = -51.4 (2)°] and the minimum and maximum bond angles at the S atom are 105.52 (12) and 118.51 (12)°, respectively. The largest of these corresponds to the O=S=O bond angle, which is usually the largest in sulfonamindes (Mughal *et al.*, 2012). An intramolecular C—H···O interaction leads to an S(6) ring.

In the crystal, inversion dimers linked by pairs of N—H···O hydrogen bonds (Table 1) generate *R*_2_^2^(8) loops (Fig. 2).

### Experimental

0.2 g of *m*-toludine was dissolved in 15 ml dichloromethane and 0.45 g of 2,5-dichlorobenzene sulfonyl chloride was added to the mixture, which was stirred at room temperature overnight. The pH was maintained at 8–9 with triethyamine. On completion of the reaction (after TLC) the pH was adjusted to 1–2 using 1*M* HCl solution. The DCM fraction was separated and the solvent evaporated at room temperature. Colourless prisms of (I) were obtained in 97% yield.

### Refinement

The H atoms were placed in calculated positions (C—H = 0.93–0.96 Å; N—H = 0.86 Å) and refined as riding. The constraint *U*_iso_(H) = 1.2*U*_eq_(C,N) or 1.5*U*_eq_(methyl C) was applied.

### Crystal data

**Table:**

C_13_H_11_Cl_2_NO_2_S	*F*(000) = 648
*M**_r_* = 316.19	*D*_x_ = 1.477 Mg m^−^^3^
Monoclinic, *P*2_1_/*n*	Mo *K*α radiation, λ = 0.71073 Å
*a* = 9.0361 (10) Å	Cell parameters from 4545 reflections
*b* = 11.6937 (11) Å	θ = 2.5–27.5°
*c* = 13.6904 (15) Å	µ = 0.60 mm^−^^1^
β = 100.588 (3)°	*T* = 296 K
*V* = 1422.0 (3) Å^3^	Prism, colourless
*Z* = 4	0.41 × 0.32 × 0.26 mm

### Data collection

**Table:**

Bruker APEXII CCD diffractometer	2019 reflections with *I* > 2σ(*I*)
Radiation source: fine-focus sealed tube	*R*_int_ = 0.033
Graphite monochromator	θ_max_ = 25.5°, θ_min_ = 2.3°
ω scans	*h* = −5→10
4252 measured reflections	*k* = −13→7
2566 independent reflections	*l* = −16→16

### Refinement

**Table:**

Refinement on *F*^2^	Primary atom site location: structure-invariant direct methods
Least-squares matrix: full	Secondary atom site location: difference Fourier map
*R*[*F*^2^ > 2σ(*F*^2^)] = 0.052	Hydrogen site location: inferred from neighbouring sites
*wR*(*F*^2^) = 0.159	H-atom parameters constrained
*S* = 1.06	*w* = 1/[σ^2^(*F*_o_^2^) + (0.1006*P*)^2^ + 0.1252*P*] where *P* = (*F*_o_^2^ + 2*F*_c_^2^)/3
2566 reflections	(Δ/σ)_max_ < 0.001
173 parameters	Δρ_max_ = 0.55 e Å^−^^3^
0 restraints	Δρ_min_ = −0.46 e Å^−^^3^

### Special details

**Table:**

Geometry. All e.s.d.'s (except the e.s.d. in the dihedral angle between two l.s. planes) are estimated using the full covariance matrix. The cell e.s.d.'s are taken into account individually in the estimation of e.s.d.'s in distances, angles and torsion angles; correlations between e.s.d.'s in cell parameters are only used when they are defined by crystal symmetry. An approximate (isotropic) treatment of cell e.s.d.'s is used for estimating e.s.d.'s involving l.s. planes.
Refinement. Refinement of *F*^2^ against ALL reflections. The weighted *R*-factor *wR* and goodness of fit *S* are based on *F*^2^, conventional *R*-factors *R* are based on *F*, with *F* set to zero for negative *F*^2^. The threshold expression of *F*^2^ > σ(*F*^2^) is used only for calculating *R*-factors(gt) *etc*. and is not relevant to the choice of reflections for refinement. *R*-factors based on *F*^2^ are statistically about twice as large as those based on *F*, and *R*- factors based on ALL data will be even larger.

### Fractional atomic coordinates and isotropic or equivalent isotropic displacement parameters (Å^2^)

**Table:**

	*x*	*y*	*z*	*U*_iso_*/*U*_eq_	
C1	0.6508 (3)	0.2746 (2)	0.5229 (2)	0.0434 (6)	
C2	0.7492 (3)	0.3602 (2)	0.5641 (2)	0.0518 (7)	
H2	0.8171	0.3473	0.6229	0.062*	
C3	0.7453 (4)	0.4650 (3)	0.5166 (3)	0.0600 (9)	
C4	0.6466 (4)	0.4856 (3)	0.4299 (3)	0.0662 (9)	
H4	0.6460	0.5562	0.3985	0.079*	
C5	0.5481 (4)	0.4010 (3)	0.3895 (3)	0.0614 (8)	
H5	0.4805	0.4145	0.3308	0.074*	
C6	0.5494 (3)	0.2971 (2)	0.4355 (2)	0.0455 (7)	
C7	0.4253 (3)	0.1890 (2)	0.66797 (19)	0.0412 (6)	
C8	0.2706 (3)	0.1896 (3)	0.6440 (2)	0.0558 (8)	
H8	0.2211	0.1433	0.5930	0.067*	
C9	0.1896 (4)	0.2589 (4)	0.6959 (3)	0.0667 (9)	
H9	0.0851	0.2590	0.6800	0.080*	
C10	0.2613 (4)	0.3283 (3)	0.7712 (3)	0.0585 (8)	
H10	0.2053	0.3753	0.8054	0.070*	
C11	0.4159 (3)	0.3281 (3)	0.7957 (2)	0.0500 (7)	
C12	0.4979 (3)	0.2580 (3)	0.7441 (2)	0.0450 (7)	
H12	0.6025	0.2573	0.7606	0.054*	
C13	0.4965 (4)	0.4020 (4)	0.8794 (3)	0.0768 (11)	
H13A	0.6001	0.3789	0.8961	0.115*	
H13B	0.4496	0.3934	0.9364	0.115*	
H13C	0.4912	0.4806	0.8588	0.115*	
S1	0.66516 (7)	0.13999 (6)	0.58357 (5)	0.0423 (3)	
N1	0.5049 (3)	0.1135 (2)	0.61442 (17)	0.0453 (6)	
H1	0.4508	0.0653	0.5765	0.054*	
O1	0.6848 (2)	0.05302 (16)	0.51318 (15)	0.0499 (5)	
O2	0.7776 (2)	0.15261 (18)	0.67029 (16)	0.0548 (6)	
Cl1	0.42138 (9)	0.19405 (7)	0.38259 (6)	0.0597 (3)	
Cl2	0.86761 (14)	0.57071 (9)	0.57067 (10)	0.0941 (4)	

### Atomic displacement parameters (Å^2^)

**Table:**

	*U*^11^	*U*^22^	*U*^33^	*U*^12^	*U*^13^	*U*^23^
C1	0.0399 (15)	0.0476 (15)	0.0414 (15)	0.0030 (12)	0.0040 (12)	−0.0048 (12)
C2	0.0420 (16)	0.0568 (18)	0.0538 (19)	−0.0010 (13)	0.0014 (13)	−0.0093 (14)
C3	0.0599 (19)	0.0472 (18)	0.076 (2)	−0.0077 (14)	0.0209 (18)	−0.0136 (16)
C4	0.087 (2)	0.0502 (19)	0.065 (2)	0.0030 (17)	0.024 (2)	0.0066 (16)
C5	0.070 (2)	0.061 (2)	0.0507 (19)	0.0029 (16)	0.0039 (16)	0.0112 (15)
C6	0.0462 (16)	0.0493 (16)	0.0382 (15)	0.0026 (12)	0.0006 (12)	−0.0016 (12)
C7	0.0460 (15)	0.0441 (15)	0.0312 (14)	−0.0006 (11)	0.0012 (11)	0.0037 (11)
C8	0.0439 (17)	0.071 (2)	0.0488 (18)	−0.0107 (14)	−0.0007 (14)	−0.0142 (15)
C9	0.0389 (17)	0.093 (3)	0.066 (2)	−0.0041 (16)	0.0035 (15)	−0.0102 (19)
C10	0.0505 (18)	0.069 (2)	0.058 (2)	0.0003 (15)	0.0148 (16)	−0.0098 (16)
C11	0.0496 (17)	0.0591 (18)	0.0402 (16)	−0.0040 (13)	0.0053 (13)	−0.0038 (13)
C12	0.0409 (15)	0.0536 (17)	0.0368 (14)	−0.0033 (12)	−0.0024 (12)	−0.0035 (12)
C13	0.070 (2)	0.085 (3)	0.072 (2)	−0.0048 (19)	0.0063 (19)	−0.038 (2)
S1	0.0405 (4)	0.0450 (4)	0.0364 (4)	0.0049 (3)	−0.0060 (3)	−0.0037 (3)
N1	0.0494 (14)	0.0462 (13)	0.0369 (13)	−0.0051 (10)	−0.0008 (10)	−0.0077 (10)
O1	0.0494 (11)	0.0508 (12)	0.0460 (11)	0.0072 (9)	0.0000 (9)	−0.0093 (9)
O2	0.0464 (12)	0.0668 (14)	0.0420 (12)	0.0069 (9)	−0.0158 (9)	−0.0045 (9)
Cl1	0.0523 (5)	0.0712 (6)	0.0468 (5)	−0.0060 (4)	−0.0143 (3)	0.0039 (3)
Cl2	0.1001 (8)	0.0623 (6)	0.1198 (10)	−0.0296 (5)	0.0204 (7)	−0.0255 (6)

### Geometric parameters (Å, º)

**Table:**

C1—C2	1.388 (4)	C8—H8	0.9300
C1—C6	1.392 (4)	C9—C10	1.376 (5)
C1—S1	1.773 (3)	C9—H9	0.9300
C2—C3	1.385 (4)	C10—C11	1.375 (5)
C2—H2	0.9300	C10—H10	0.9300
C3—C4	1.369 (5)	C11—C12	1.384 (4)
C3—Cl2	1.732 (3)	C11—C13	1.510 (4)
C4—C5	1.377 (5)	C12—H12	0.9300
C4—H4	0.9300	C13—H13A	0.9600
C5—C6	1.367 (4)	C13—H13B	0.9600
C5—H5	0.9300	C13—H13C	0.9600
C6—Cl1	1.734 (3)	S1—O2	1.421 (2)
C7—C8	1.376 (4)	S1—O1	1.434 (2)
C7—C12	1.384 (4)	S1—N1	1.611 (3)
C7—N1	1.425 (4)	N1—H1	0.8562
C8—C9	1.374 (5)		
			
C2—C1—C6	119.0 (3)	C10—C9—H9	119.6
C2—C1—S1	117.6 (2)	C11—C10—C9	120.0 (3)
C6—C1—S1	123.4 (2)	C11—C10—H10	120.0
C3—C2—C1	119.2 (3)	C9—C10—H10	120.0
C3—C2—H2	120.4	C10—C11—C12	119.4 (3)
C1—C2—H2	120.4	C10—C11—C13	120.8 (3)
C4—C3—C2	121.3 (3)	C12—C11—C13	119.8 (3)
C4—C3—Cl2	120.5 (3)	C11—C12—C7	120.3 (3)
C2—C3—Cl2	118.1 (3)	C11—C12—H12	119.8
C3—C4—C5	119.4 (3)	C7—C12—H12	119.8
C3—C4—H4	120.3	C11—C13—H13A	109.5
C5—C4—H4	120.3	C11—C13—H13B	109.5
C6—C5—C4	120.3 (3)	H13A—C13—H13B	109.5
C6—C5—H5	119.9	C11—C13—H13C	109.5
C4—C5—H5	119.9	H13A—C13—H13C	109.5
C5—C6—C1	120.8 (3)	H13B—C13—H13C	109.5
C5—C6—Cl1	118.5 (2)	O2—S1—O1	118.51 (12)
C1—C6—Cl1	120.7 (2)	O2—S1—N1	109.86 (13)
C8—C7—C12	119.8 (3)	O1—S1—N1	105.52 (12)
C8—C7—N1	117.9 (3)	O2—S1—C1	106.17 (13)
C12—C7—N1	122.3 (3)	O1—S1—C1	108.81 (13)
C9—C8—C7	119.5 (3)	N1—S1—C1	107.55 (13)
C9—C8—H8	120.2	C7—N1—S1	125.4 (2)
C7—C8—H8	120.2	C7—N1—H1	115.7
C8—C9—C10	120.9 (3)	S1—N1—H1	114.1
C8—C9—H9	119.6		
			
C6—C1—C2—C3	−0.7 (4)	C9—C10—C11—C12	0.1 (5)
S1—C1—C2—C3	177.4 (2)	C9—C10—C11—C13	−178.8 (4)
C1—C2—C3—C4	−0.1 (5)	C10—C11—C12—C7	0.3 (5)
C1—C2—C3—Cl2	179.1 (2)	C13—C11—C12—C7	179.3 (3)
C2—C3—C4—C5	0.6 (5)	C8—C7—C12—C11	−0.4 (4)
Cl2—C3—C4—C5	−178.6 (3)	N1—C7—C12—C11	−178.4 (3)
C3—C4—C5—C6	−0.2 (5)	C2—C1—S1—O2	3.2 (3)
C4—C5—C6—C1	−0.6 (5)	C6—C1—S1—O2	−178.7 (2)
C4—C5—C6—Cl1	179.1 (3)	C2—C1—S1—O1	−125.3 (2)
C2—C1—C6—C5	1.1 (4)	C6—C1—S1—O1	52.7 (3)
S1—C1—C6—C5	−176.9 (2)	C2—C1—S1—N1	120.8 (2)
C2—C1—C6—Cl1	−178.6 (2)	C6—C1—S1—N1	−61.1 (3)
S1—C1—C6—Cl1	3.4 (4)	C8—C7—N1—S1	144.7 (2)
C12—C7—C8—C9	0.1 (5)	C12—C7—N1—S1	−37.2 (4)
N1—C7—C8—C9	178.2 (3)	O2—S1—N1—C7	63.7 (2)
C7—C8—C9—C10	0.4 (6)	O1—S1—N1—C7	−167.5 (2)
C8—C9—C10—C11	−0.5 (6)	C1—S1—N1—C7	−51.4 (2)

### Hydrogen-bond geometry (Å, º)

**Table:**

*D*—H···*A*	*D*—H	H···*A*	*D*···*A*	*D*—H···*A*
N1—H1···O1^i^	0.86	2.09	2.946 (3)	178
C12—H12···O2	0.93	2.50	3.141 (3)	126

Symmetry code: (i) −*x*+1, −*y*, −*z*+1.
